# Ex Vivo Evaluation of the Fit of Matched Gutta Percha Points in Human Root Canals Prepared With the Corresponding Nickel‐Titanium Files

**DOI:** 10.1111/aej.70000

**Published:** 2025-08-08

**Authors:** Samuel Deng, Paul V. Abbott

**Affiliations:** ^1^ UWA Dental School The University of Western Australia Nedlands WA Australia

**Keywords:** fit, matched gutta percha, micro‐CT, rotary file, unfilled space

## Abstract

The aim of this study was to evaluate the fit of matched gutta‐percha (GP) points in tooth root canals after preparation with their corresponding rotary files. Single canal human tooth roots were matched according to root canal volume and eccentricity. Forty‐five roots were divided into three experimental groups (*n* = 15) and sequentially prepared with either SybronEndo TF Adaptive SM2, ProTaper Next X2 or ProTaper Ultimate F2 files to working length. The corresponding GP points were placed into the prepared root canals without cement and scanned with micro‐computed tomographic imaging. The unfilled volume and unfilled areas at 1, 3, 5 and 7 mm from working length were evaluated. Over one‐third of the root canal space remained unfilled in all groups. There was no significant difference between unfilled areas measured at the different distances. Unprepared and unfilled spaces remained in root canals after preparation with rotary files when filled with the corresponding GP points.

## 1 Introduction

While gutta‐percha (GP) is the gold standard core root canal filling material, there will inevitably be voids left in the root canal space. Endodontic cements are used to fill these spaces, but they may contract upon setting and they can contain chemicals that leach out over time [1, 2, 3]. This can lead to voids in the root canal filling, providing an environment to facilitate bacterial growth and a route for bacteria and their by‐products to reach the periapical tissues [4, 5]. Therefore, it is important that the GP dimensionally matches the prepared root canal to minimise the space that needs to be filled with cement.

As nickel‐titanium (Ni‐Ti) rotary instruments can predictably shape the root canals, manufacturers also make GP points of corresponding shape. This has popularised the single point obturation (SPO) technique, which uses a matched GP point to fill the prepared root canal space without needing to place accessory points or compact the GP. Manufacturers claim this will accurately fill the prepared space, ensuring the maximum amount of GP and minimal cement.

The size and taper of endodontic files are standardised according to the International Organisation of Standardisation (ISO 3630‐1) [6]. In addition, GP points are manufactured according to the International Organisation of Standardisation (ISO 6877) and the American National Standards Institute/American Dental Association (ANSI/ADA Specification No. 78) to ensure they match the corresponding files [7, 8].

Despite standardisation, there is reported variation in the dimensions of files and GP points. Rotary systems such as ProFile (Dentsply Maillefer, Ballaigues, Switzerland), RaCe (FKG Dentaire, La Chaux‐de‐Fonds, Switzerland), and TF Adaptive files (SybronEndo, KaVo Kerr, California, USA) have been reported to vary outside the accepted range [9]. In addition, GP points have considerable variability but generally meet the required specifications due to the large range permitted within the standards [10].

Within rotary systems, files and GP points of the supposedly same size show significantly different dimensions. ProTaper Next and WaveOne (Dentsply Maillefer, Ballaigues, Switzerland) have significantly larger matched GP points when compared with the corresponding rotary file [11]. EndoSequence (Busa, Brasseler, Georgia, USA), ProTaper and K3 (KaVo Kerr, California, USA) files were all significantly smaller than their corresponding GP points, with EndoSequence files falling outside the ISO specified range. The taper was also significantly different, with ProTaper files and GP points much larger than the specified 0.04 taper [12]. The MTwo (VDW, Munich, Germany) rotary system also had significantly larger files when measured against their matched GP points [13].

Producing files and GP points at the extreme but tolerable dimensions can affect the length to which the root canal is prepared and filled. A cross‐sectional study revealed 17.20%–33.33% of single‐point root canal fillings were placed short of the desired length, and 0.94%–6.55% were filled beyond the apical foramen [14]. The greater number of underfilled root canals compared to overextended root fillings may be due to the tendency of the matched GP points to be larger than the corresponding files of the same size. Experienced clinicians may be able to identify and correct the size discrepancies by either adjusting the GP point or choosing a different one. However, this will cause frustration and waste time during treatment, especially when the appeal of the SPO technique is its claimed simplicity and efficiency.

While the SPO technique is claimed to be comparable to lateral compaction, experiments have used simulated canals in acrylic blocks [15]. The human root canal morphology is complex, and therefore, root canal fillings cannot be adequately evaluated using acrylic blocks. In other experiments [16, 17], sectioned tooth roots were viewed at different levels. However, irrigation during sectioning may wash away the cement, and the diamond saw used to cut the samples may deform the GP. Comparisons of these root filling techniques have also been made based on two‐dimensional radiographic appearance [18]. However, a root canal filling is a three‐dimensional object, and it cannot be assessed accurately with 2‐D imaging.

More recently, microcomputed tomography (micro‐CT) imaging has been used to evaluate root canal fillings [19]. The advantages of using micro‐CT are that samples can be viewed and analysed in three dimensions without damaging the teeth or distorting the GP [20]. They can also be viewed and compared at different stages of the treatment—for example, before and after the root canal filling.

Given the appeal of the SPO technique, it is fast becoming widely utilised. However, current research has only evaluated voids in the root canal filling (GP and cement), and there is minimal evidence evaluating the fit of the GP point in the prepared root canals. As rotary files and the corresponding GP points may not follow the accepted ISO range and may not match dimensions with one another, even within the same rotary system, their ability to adequately fill the complex human root canal may be affected. Therefore, the aim of this study was to assess how accurately the matched GP points fill root canals prepared with their corresponding Ni‐Ti rotary files.

## 2 Materials and Method

Ethics Exemption was approved by the University of Western Australia Human Research Ethics Office on the 29th April 2022—ET000284. Human teeth used in this research were selected from the Tooth Bank of the Oral Health Centre of Western Australia. General consent to use these teeth for education and/or research had been obtained from the patients prior to extraction.

Seventy‐two mature human single canal premolars, incisors, canines, palatal roots of upper molars and distal roots of lower molars were collected from the Tooth Bank. The roots were radiographed through 2‐D imaging in both frontal and sagittal directions to confirm the presence of one canal and to exclude roots with multiple canals, accessory canals, an open apical foramen, a large apical canal or fractures. Fifteen roots did not meet the inclusion criteria. The remaining 57 roots were mounted perpendicularly in a styrofoam box and scanned in a micro‐CT machine (In vivo Micro‐CT Bruker Skyscan 1176 micro‐CT at an x‐ray voltage of 70 kV, a current of 114 mA, a voxel size of 18 μm, scanned at 360° rotation with a 0.5° step, frame averaging of 2 and a 1.0 mm aluminium filter), and the images were reconstructed using NRecon (v.1.6.9.8 software; Bruker MicroCT). The scans were transferred to DataViewer (v.1.5.0 software; Bruker MicroCT) to isolate the axial view of the samples, and then they were transferred to the micro‐CT software CTAn (v.1.15 software; Bruker MicroCT) to evaluate the root canal configuration, including the size of the apical part of the canal and the apical foramen.

**FIGURE 1:**
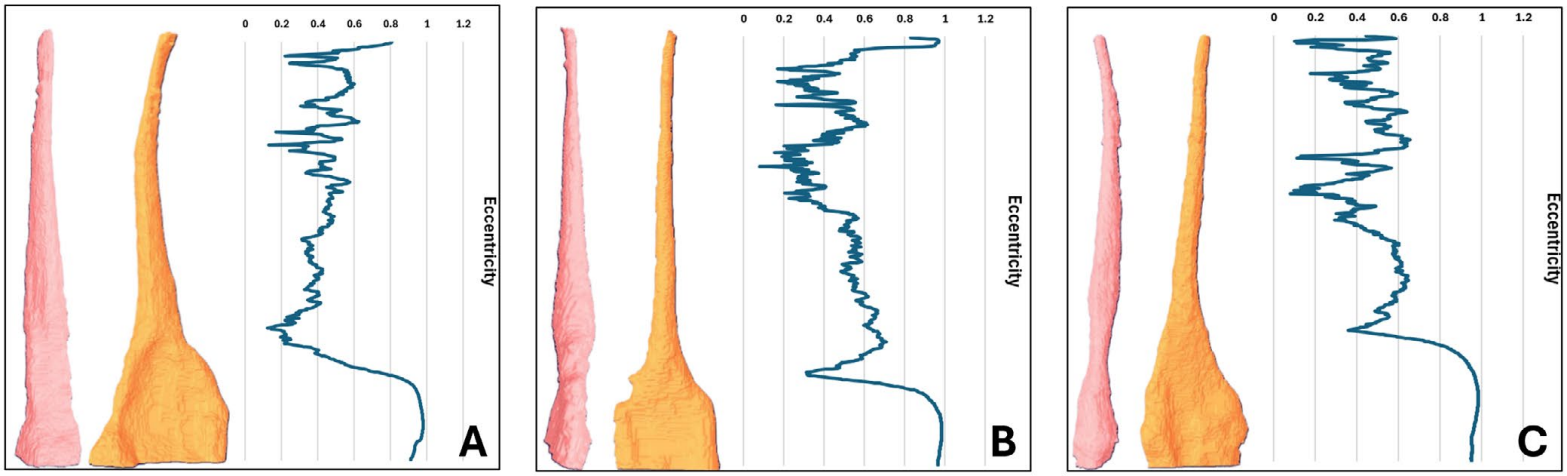
Allocation into the different experimental groups was based on similar root canal morphology. This figure shows the frontal and interproximal views of the root canals of three permanent upper premolars after micro‐CT scanning and 3‐D reconstruction. For each root canal, the eccentricity value was calculated at each cross‐section and graphed (shown next to the 3‐D model). All samples were matched according to similar graphs and allocated to the experimental groups.

After scanning, a further 10 roots were excluded. The remaining 47 roots were allocated into experimental groups based on the method of De‐Deus et al. [21] where root canal volume and aspect ratios were measured from the cemento‐enamel junction to the root apex. The aspect ratio measures canal circularity at a particular cross‐section by calculating a ratio of the largest and smallest diameters of an oval. In this study, eccentricity was measured instead of the aspect ratio for each cross‐section. Eccentricity measures how round a shape is, with a higher eccentricity indicating an elongated object and a lower eccentricity indicating a circular shape. Eccentricity values at each cross‐section for all samples were plotted on a graph. Samples were matched according to similar canal volumes and eccentricity graph shapes, and then they were allocated to three different groups to ensure an even distribution of canal morphologies. Figure 1 shows an example of three roots with similar morphologies. A power calculation based on previous work [22] with α = 0.05 and power = 0.80 for independent sample groups indicated a sample size of at least 12 was required.

Fifteen roots were allocated to each experimental group. Access was gained to each canal and working lengths (WL) were determined using a size #10 H‐file (Kerr Dental, Orange, CA, USA). When the file tip reached the apical foramen, 1.0 mm was subtracted. Initially, a size #15 H‐file was used to obtain the WL and then the canal preparation was completed according to the following protocols:

### 2.1 TF Adaptive Group

Root canal preparation was completed using the TF Adaptive rotary file system (SybronEndo) SM1 (20/0.04) and SM2 (25/0.06) files to the predetermined working length using pecking motions. The SybronEndo Elements Motor (KaVo Kerr, Glendora, CA, USA) with the Adaptive Motion technology feature was used.

### 2.2 ProTaper Next Group

Root canal preparation was completed using the ProTaper Next rotary system (Dentsply Maillefer, Ballaigues, Switzerland) X1 (17/0.04) and X2 (25/0.06) files to the predetermined working length using thrpecking motions three times. The Tri‐Auto ZX2 (J. Morita Corp, Osaka, Japan) rotary handpiece was used and set to a speed of 400 RPM with a torque of 4.0 Ncm and continuous clockwise rotation.

### 2.3 ProTaper Ultimate Group

Root canal preparation was completed using the ProTaper Ultimate rotary system (Dentsply Maillefer) Slider (16/0.02), Shaper (20/0.04), F1 (20/0.07) and F2 (25/0.08) files to the predetermined working length using pecking motions three times. The Tri‐Auto ZX2 rotary handpiece was used and set to a speed of 400 RPM with a torque of 4.0 Ncm and continuous clockwise rotation.

All canals were irrigated with 15% EDTA/C (15% EDTA and 0.85% Cetrimide; DentaLife Pty. Ltd., Ringwood, VIC, Australia) and dried using paper points.

**FIGURE 2:**
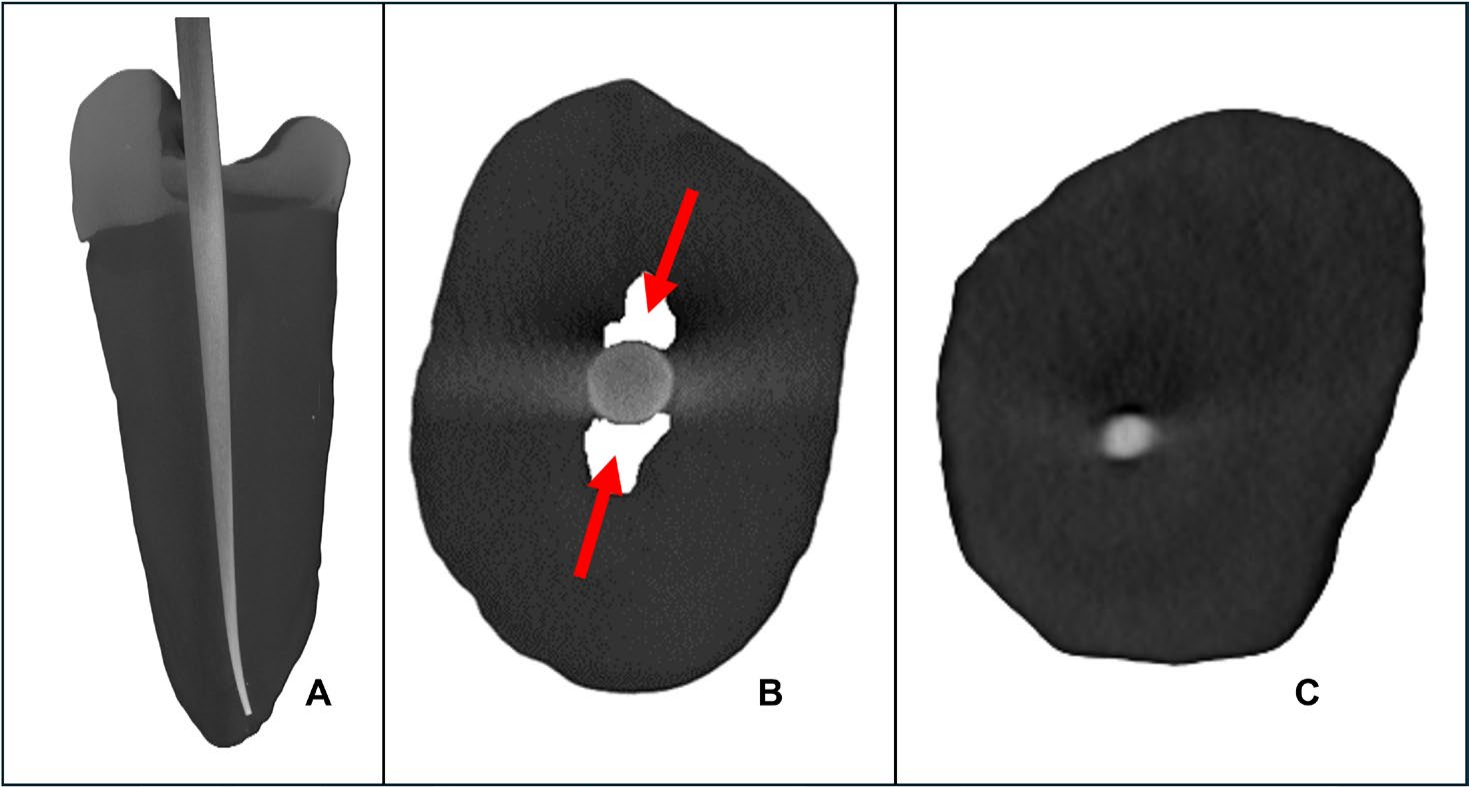
(A) Raw micro‐CT images of a lower premolar from the ProTaper Next group with its root canal prepared with a rotary file and filled with the matched GP point. (B) A cross‐section of the sample in the coronal part of the root with large unfilled areas within the canal (arrows). (C) A cross‐section of the same tooth but in the apical portion where the GP has filled most of the prepared canal.

Corresponding matched GP points were placed into the prepared canals (without cement) and micro‐CT scanning with the same parameters was performed. The images were again reconstructed using NRecon. The axial view was isolated from DataViewer, and the scans were transferred to CTAn for analysis. Figure 2 illustrates the raw micro‐CT images of a lower premolar after root canal preparation and filling with the matched GP point. Three‐dimensional analysis of the samples was completed to calculate the percentage of the unfilled space of the canal, measuring from the level of the labial/buccal CEJ to the working length. Cross‐sections of 1, 3, 5 and 7 mm from the working length were obtained, and the percentage of the unfilled area at these levels was evaluated to assess the fit of the GP point in the apical half of the canals.

Numerical data were analysed using the Shapiro–Wilk's test and were normally distributed. Volume data were analysed using one‐way ANOVA followed by Tukey's post hoc test. Area data were analysed using a two‐way mixed model ANOVA followed by the Bonferroni post hoc test. Significance level was *p* < 0.05 for all tests. Statistical analysis was performed with R statistical analysis software version 4.3.1 for Windows (Microsoft, Albuquerque, New Mexico, USA).

## 3 Results

Results of intergroup comparisons of unfilled volumes are presented in Table 1. These show that at least one third of the root canals remained unfilled after shaping and filling using the tested rotary systems. The TF Adaptive group had significantly higher unfilled volumes than both ProTaper systems (*p* < 0.001).

**TABLE 1:** Intergroup comparison of the mean ± standard deviation of the unfilled volumes (%).

*Unfilled volume (%)*			*f*	*p*
*TF Adaptive*	*ProTaper Next*	*ProTaper Ultimate*		
56.06 ± 14.48^A^	36.76 ± 10.58^B^	35.84 ± 9.98^B^	13.93	< 0.001*

*Note:* Values with different superscript letters within the same horizontal row are significantly different.

* Significant (*p* < 0.05).

**FIGURE 3:**
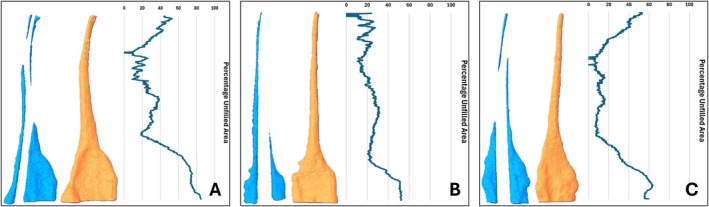
3‐D reconstruction of the unfilled area after the root canal was prepared with rotary files and the corresponding GP point was placed compared with the 3‐D model of the preoperative root canal. The percentage of the unfilled area at each cross section was also graphed (next to the model). (A) TF Adaptive. (B) ProTaper Next. (C) ProTaper Ultimate.

Table 2 shows that the TF Adaptive group had significantly higher unfilled areas when compared to the other systems, regardless of the distance from the WL (*p* < 0.001). There was no significant difference between values measured at different distances from the working length within each experimental group (*p* = 0.301). Figure 3 illustrates the location and extent of the unfilled areas in the three premolars from Figure 1, with the respective graph of the percentage of unfilled area at each cross‐section.

**TABLE 2:** Inter and intragroup comparisons of the mean ± standard deviation of the unfilled areas (%).

Distance from the working length	Unfilled area (%)			*t*	*p*
	TF Adaptive	ProTaper Next	ProTaper Ultimate		
1 mm	48.30 ± 14.84^Aa^	32.32 ± 2.74^Ba^	36.20 ± 12.47^Ba^	6.72	0.004*
3 mm	40.48 ± 10.01^Aa^	33.52 ± 7.91^Ba^	29.33 ± 4.26^Ba^	6.79	0.003*
5 mm	47.29 ± 12.69^Aa^	31.02 ± 4.65^Ba^	34.36 ± 8.81^Ba^	11.22	< 0.001*
7 mm	50.16 ± 16.47^Aa^	32.19 ± 3.79^Ba^	31.28 ± 10.54^Ba^	12.64	< 0.001*
*f*	2.18	0.78	1.44		
*p*	0.106	0.512	0.251		

*Note:* Values with different upper and lowercase superscript letters within the same horizontal row and vertical column respectively are significantly different.

* Significant (*p* < 0.05).

## 4 Discussion

Bacterial penetration through a root‐filled tooth can occur through the cement‐dentine interface, the cement‐GP interface, through voids or through the dentinal tubules [23]. Gutta‐percha does not change shape or size, and it is impervious to bacteria. However, endodontic cements can contract, degrade and may be soluble, leading to loss of integrity of the root canal filling [2]. Therefore, the focus should be on maximising the amount of GP in the prepared root canal and minimising the amount of cement.

The aim of the current study was not to compare the shaping and filling ability of the different rotary systems but to evaluate how well the matched GP points filled the prepared root canals. The ProTaper systems were chosen because they are very commonly used, and TF Adaptive because it is a reciprocating system. The results show that over a third of the root canal remained unfilled after shaping with the corresponding rotary files and placement of the matched GP. The TF Adaptive group had more unfilled area at all levels, as well as overall, compared with both the ProTaper Next and ProTaper Ultimate groups. The manufacturers of all files tested claim that they have an ISO size of 25 at the apex. The TF Adaptive SM2 and ProTaper Next X2 have a taper of 6%, while the ProTaper Ultimate F2 has an apical taper of 8%.

**FIGURE 4:**
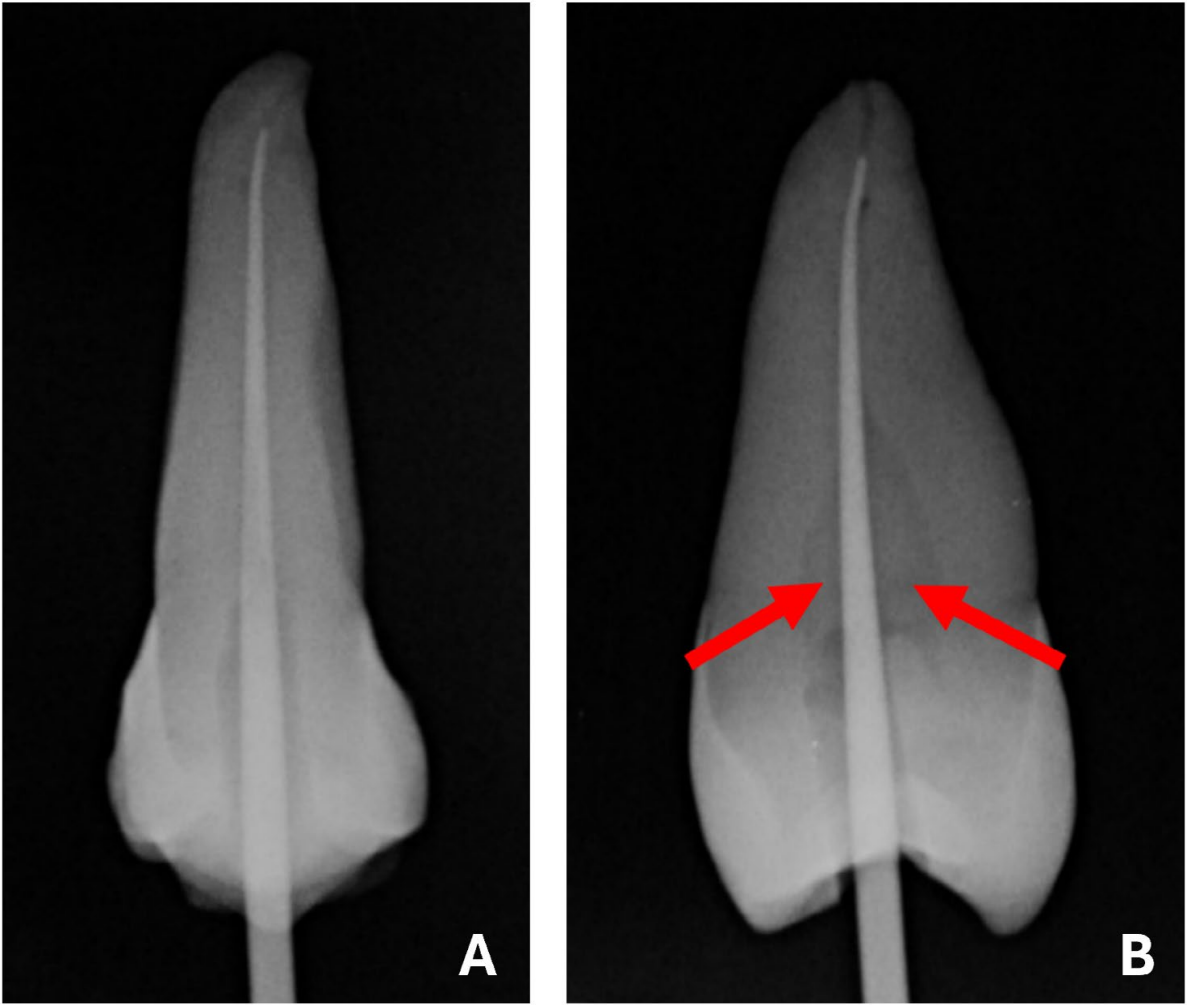
An upper premolar that has been prepared with a rotary file and filled with the matched GP point from the ProTaper Next rotary system. (A) The buccal view suggests that the canal has been adequately filled with the single GP point. (B) An interproximal view of the same tooth which reveals large spaces between the GP and the dentine walls (arrows), confirming poor filling of the root canal with the matched GP point.

Root canals can appear filled with the corresponding matched GP point in 2‐D imaging, but large spaces may remain between the GP and the dentinal walls. Figure 4 shows an upper premolar that has been prepared with rotary files and filled with its matched GP point. The radiograph from the buccal view would lead many clinicians to believe that the canal has been adequately filled with GP; however, the radiograph taken from the interproximal view reveals large unfilled spaces. Clinically, only the frontal view can be visualised. Therefore, clinicians may be overestimating the amount of GP within the root canal space.

There was no significant difference between the percentage of unfilled areas at 1, 3, 5 and 7 mm from the working length for all systems tested. This indicates that as the cross‐sectional size of the prepared canal gets larger from the apical end to the coronal end, there is a proportional increase in the GP size to fill the space. However, some teeth have root canals with a wide flare in the buccal‐palatal plane that is coronal to these levels, which is highlighted in Figures 2 and 3. Human teeth tend to have root canals with a rounder cross‐section apically, and an oval cross‐section coronally [24]. This coronal area will remain unfilled or will be filled with a large amount of cement if the SPO obturation technique is used.

In some samples, the GP point did not reach the working length, while in others, the GP point extended further than the working length. However, the analysis was performed to the predetermined working length because practitioners can cut off the extruded GP in clinical situations, provided they notice the GP point is seated too far into the canal. Smaller GP points will be able to easily reach the working length, but they may be extruded into the periapical tissues, which could reduce the success of root canal treatment from 94% to 76% after 8–10 years [25]. Furthermore, premature binding of the GP point against the root canal walls will lead to a short root canal filling, also potentially reducing the success of the treatment to 68% [25]. Clinicians should be aware that GP points may need adjustments, or another point may need to be selected to reach the desired working length.

The human tooth root canal morphology is complex, with the possibility of lateral canals, accessory canals, apical deltas, C‐shaped canals, oval‐shaped canals and isthmuses. Therefore, root canal fillings cannot be adequately evaluated using acrylic blocks. This important factor has been previously highlighted, where the percentage of gutta‐percha‐filled areas (PGFA) in prepared curved acrylic blocks ranged from 94% and 100% while in the MB root of extracted maxillary first molars, it was between 72% and 96% [15]. Therefore, it is imprudent to conclude that rotary files can prepare all root canals into a predictable shape that can be completely filled with the corresponding matched GP point.

A study concluded that with the SPO technique, the PGFA in extracted teeth varied from 61.1% to 83.6%, which was significantly less compared with lateral compaction, which had a PGFA between 80.05% and 93% [26]. The authors also noted that in the apical 4 mm of the tooth, cold lateral compaction using a matched master point had a lower PGFA compared to cold lateral compaction with a standard hand‐rolled non‐matched master point of the respective size. While not significant, this could be attributed to improper seating of the matched master point in the prepared space. In addition, the premature binding in the more coronal parts of the canal (as a result of the increased taper) may inhibit the penetration of the spreader to the apical third for compaction [27]. A prepared root canal that has less volume of GP may lead to voids or pooling of the cement in the root canal filling [15].

Endodontic retreatment is more challenging when there is more endodontic cement. If previous root canal filling material is left within the canals, bacteria can be found on the rough surfaces and between the dentinal walls and the root canal filling material, where they can colonise within the dentinal tubules [23]. If all the root canal filling material is not removed, the microflora in these areas will potentially be protected from medicaments and irrigants. This can lead to incomplete disinfection of the root canal system, contributing to persistent periapical inflammation. While it is impossible to completely remove the previous root canal filling material, [28] clinicians should aim to remove as much of it as possible.

Removal of GP is enhanced with the help of solvents such as eucalyptus oil and chloroform, which can soften the GP. However, these solvents show minimal effects on most endodontic cements [29]. Complete removal of epoxy resin cements or bioceramic cements in canals filled with the SPO technique cannot be achieved. Furthermore, cement in the apical portion of the root canal can inhibit the ability to reach the working length [30]. With solvents having minimal effects, hand files, rotary files and ultrasonic instruments are the most effective instruments to remove endodontic cements. However, excessive instrumentation may lead to ledging, perforation or unnecessary canal enlargement.

A limitation of this study was the large variation in root canal anatomy, size and shape. While pair matching was performed with micro‐CT to limit selection bias, some variation still exists. The quality of the reconstructed micro‐CT images was affected by the GP point, which has a high attenuation. This made it difficult to accurately identify the empty spaces within the root canal in some samples. All canals were prepared to an apical size of 25 and a taper of at least 6% in order to standardise the preparation size and GP points used. However, a larger apical preparation size may have been more suitable for some samples. Also, only roots with a single canal were included in this experiment. Roots with multiple canals have narrower root canals, and this may result in a smaller unfilled volume after preparation with rotary files and filling with the matched GP point. Future studies should investigate the fit of different‐sized matched GP points in a variety of prepared human tooth root canals.

This study has revealed inadequate fit of matched GP points when used with the SPO technique after preparation with the corresponding rotary file. Furthermore, these unfilled spaces may be undetected during the root canal filling. This technique may be more appropriate for small diameter canals. Oval‐shaped and larger diameter root canals would require excessive preparation for this to be effective. Therefore, different root canal preparation and root filling techniques would be more suitable in these cases.

## Author Contributions

All authors have contributed significantly and are in agreement with the manuscript.

## Disclosure

The authors have nothing to report.

## Conflicts of Interest

The authors declare no conflicts of interest.

## Supporting information


**Data S1:** Supporting Information.

## Data Availability

The data that support the findings of this study are available from the corresponding author upon reasonable request.

## References

[1] W. Geurtsen and G. Leyhausen , “Biological Aspects of Root Canal Filling Materials ‐ Histocompatibility, Cytotoxicity, and Mutagenicity,” Clinical Oral Investigations 1, no. 1 (1997): 5–11. 9552811 10.1007/s007840050002

[2] R. B. Kazemi , K. E. Safavi , and L. S. W. Spångberg , “Dimensional Changes of Endodontic Sealers,” Oral Surgery, Oral Medicine, and Oral Pathology 76 (1993): 766–771. 8284084 10.1016/0030-4220(93)90050-e

[3] Y. Elyassi , A. T. Moinzadeh , and C. J. Kleverlaan , “Characterization of Leachates From 6 Root Canal Sealers,” Journal of Endodontics 45 (2019): 623–627. 30905572 10.1016/j.joen.2019.01.011

[4] G. Sundqvist , “Bacteriological Studies of Necrotic Dental Pulps,” (1976), Umeå University.

[5] T. N. Nguyen , “Obturation of the Root Canal System,” in Pathways of the Pulp, 6th ed., ed. S. Cohen and R. C. Burns (Mosby Year Book, 1994), 219–271.

[6] International Organization for Standardization , “Dentistry—Endodontic Instruments Part 1: General Requirements,” (2019), https://www.iso.org/standard/75260.html.

[7] ANSI , “American National Standards Institute/American Dental Association Specification No. 78 for Dental Obturating Cones,” (2013), https://webstore.ansi.org/standards/ada/ansiada782013.

[8] International Organization for Standardization , “Dental Root‐Canal Obturating Points,” (1995), https://www.iso.org/standard/72350.html.

[9] K. W. Kim , K. M. Cho , S. H. Park , K. Y. Choi , B. Karabucak , and J. W. Kim , “A Comparison of Dimensional Standard of Several Nickel‐Titanium Rotary Files,” Restorative Dentistry & Endodontics 39 (2014): 7–11. 24516823 10.5395/rde.2014.39.1.7 PMC3916510

[10] K. P. Cunningham , M. P. Walker , J. C. Kulild , and J. T. Lask , “Variability of the Diameter and Taper of Size #30, 0.04 Gutta‐Percha Cones,” Journal of Endodontics 32 (2006): 1081–1084. 17055911 10.1016/j.joen.2006.06.007

[11] H. Mirmohammadi , M. Sitarz , and H. Shemesh , “Intra‐Manufacture Diameter Variability of Rotary Files and Their Corresponding Gutta‐Percha Cones Using Laser Scan Micrometre,” Iranian Endodontic Journal 13 (2018): 159–162. 29707008 10.22037/iej.v13i2.14710 PMC5911287

[12] M. B. Chesler , P. A. Tordik , G. M. Imamura , and G. G. Goodell , “Intra‐Manufacturer Diameter and Taper Variability of Rotary Instruments and Their Corresponding Gutta‐Percha Cones,” Journal of Endodontics 39 (2013): 538–541. 23522553 10.1016/j.joen.2012.12.029

[13] A. A. Salles , C. B. Cord , T. S. Sonnemann , T. A. de Melo , L. E. Irala , and E. P. de Oliveira , “Comparative Analysis of the Diameter of MTwo System Gutta‐Percha Points in Relation to Their Corresponding Instruments,” Revista Brasileira de Odontologia 10 (2013): 49–55.

[14] G. Gavini , G. T. M. Candeiro , F. Potgornik Ferreira , et al., “Retrospective Study of Endodontic Treatment Performed by Undergraduate Students Using Reciprocating Instrumentation and Single‐Cone Obturation,” Journal of Dental Education 86 (2022): 751–758. 35061917 10.1002/jdd.12884

[15] M. P. J. Gordon , R. M. Love , and N. P. Chandler , “An Evaluation of .06 Tapered Gutta‐Percha Cones for Filling of .06 Taper Prepared Curved Root Canals,” International Endodontic Journal 38 (2005): 87–96. 15667630 10.1111/j.1365-2591.2004.00903.x

[16] E. Schaefer , M. Koester , and S. Buerklein , “Percentage of Gutta‐Percha–Filled Areas in Canals Instrumented With Nickel‐Titanium Systems and Obturated With Matching Single Cones,” Journal of Endodontics 39 (2013): 924–928. 23791265 10.1016/j.joen.2013.04.001

[17] C. Romania , P. Beltes , C. Boutsioukis , and C. Dandakis , “Ex‐Vivo Area‐Metric Analysis of Root Canal Obturation Using Gutta‐Percha Cones of Different Taper,” International Endodontic Journal 42 (2009): 491–498. 19460000 10.1111/j.1365-2591.2008.01533.x

[18] P. Hörsted‐Bindslev , M. A. Andersen , M. F. Jensen , J. H. Nilsson , and A. Wenzel , “Quality of Molar Root Canal Fillings Performed With the Lateral Compaction and the Single‐Cone Technique,” Journal of Endodontics 33 (2007): 468–471. 17368341 10.1016/j.joen.2006.12.016

[19] M. R. Kalantar Motamedi , A. Mortaheb , M. Zare Jahromi , and B. E. Gilbert , “Micro‐CT Evaluation of Four Root Canal Obturation Techniques,” Scanning 1 (2021): 6632822. 33717394 10.1155/2021/6632822 PMC7932785

[20] K. Orhan , R. Jacobs , B. Celikten , et al., “Evaluation of Threshold Values for Root Canal Filling Voids in Micro‐CT and Nano‐CT Images,” Scanning 2018, no. 1 (2018): 9437569. 30116470 10.1155/2018/9437569 PMC6079325

[21] G. De‐Deus , M. Simões‐Carvalho , F. G. Belladonna , et al., “Creation of Well‐Balanced Experimental Groups for Comparative Endodontic Laboratory Studies: A New Proposal Based on Micro‐CT and In Silico Methods,” International Endodontic Journal 53 (2020): 974–985. 32159857 10.1111/iej.13288

[22] I. D. Capar , H. Ertas , E. Ok , and H. Arslan , “Comparison of Single Cone Obturation Performance of Different Novel Nickel‐Titanium Rotary Systems,” Acta Odontologica Scandinavica 72 (2014): 537–542. 24460041 10.3109/00016357.2013.876554

[23] S. Kwang and P. Abbott , “The Presence and Distribution of Bacteria in Dentinal Tubules of Root Filled Teeth,” International Endodontic Journal 47 (2014): 600–610. 24111689 10.1111/iej.12195

[24] M. K. Wu , A. R'Oris , D. Barkis , and P. R. Wesselink , “Prevalence and Extent of Long Oval Canals in the Apical Third,” Oral Surgery, Oral Medicine, Oral Pathology, Oral Radiology, and Endodontology 89 (2000): 739–743. 10.1067/moe.2000.106344 10846130

[25] U. Sjögren , B. Hagglund , G. Sundqvist , and K. Wing , “Factors Affecting the Long‐Term Results of Endodontic Treatment,” Journal of Endodontics 16 (1990): 498–504. 2084204 10.1016/S0099-2399(07)80180-4

[26] E. Schäfer , B. Nelius , and S. Bürklein , “A Comparative Evaluation of Gutta‐Percha Filled Areas in Curved Root Canals Obturated With Different Techniques,” Clinical Oral Investigations 16 (2011): 225–230. 21249509 10.1007/s00784-011-0509-z

[27] M. Tanomaru‐Filho , D. V. B. Trindade , L. T. de Almeida , C. G. Espir , I. Bonetti‐Filho , and J. M. Guerreiro‐Tanomaru , “Effect of ProTaper and Reciproc Preparation and Gutta‐Percha Cone on Cold Lateral Compaction,” Journal of Conservative Dentistry 19 (2016): 410–413. 27656057 10.4103/0972-0707.190015 PMC5026098

[28] J. F. Schirrmeister , K. M. Meyer , P. Hermanns , M. J. Altenburger , and K. T. Wrbas , “Effectiveness of Hand and Rotary Instrumentation for Removing a New Synthetic Polymer‐Based Root Canal Obturation Material (Epiphany) During Retreatment,” International Endodontic Journal 39 (2006): 150–156. 16454796 10.1111/j.1365-2591.2006.01066.x

[29] K. I. Zhekov and V. P. Stefanova , “Retreatability of Bioceramic Endodontic Sealers: A Review,” Folia Medica 62 (2020): 258–264. 32666747 10.3897/folmed.62.e47690

[30] H. C. Baranwal , N. Mittal , R. Garg , J. Yadav , and P. Rani , “Comparative Evaluation of Retreatability of Bioceramic Sealer (BioRoot RCS) and Epoxy Resin (AH Plus) Sealer With Two Different Retreatment Files: An In Vitro Study,” Journal of Conservative Dentistry 24 (2021): 88–93. 34475687 10.4103/jcd.jcd_657_20 PMC8378486

